# A Remarkable Case

**Published:** 1885-05

**Authors:** Frank H. Nichols

**Affiliations:** Atlanta, Ga.


### A REMARKABLE CASE.

BY FRANK H. NICHOLS, M. D., ATLANTA, GA.

We copy the following from our notes and memoranda of practice:

In October, 1879, was called to see Mrs. R., living on the Chattahoochee river, aged 28 years, of robust constitution, and mother of four children, the youngest three years old.

We learned that Mrs. R. had been complaining two days, with rigors and some fever, with increasing pain in head and back, and cramps in the lower extremities. Upon examination, we found the head drawn backward, the pulse rapid, the breathing hurried at times, attended with great restlessness.

The family attributed her sickness to having her feet wet while scouring and cleaning the house the day before she was taken sick. She was seven months gone in pregnancy. Our diagnosis was carefully made out, and the danger fully explained to the family.

The treatment was warm baths, warm application to the feet, cold to head, counter-irritation and hot anodyne application to the spine, with a mild laxative to open the bowels. The case was watched with sedulous care. On the morning of the third day, the symptoms ameliorated, and we felt encouraged to hope recovery would take place. That night the patient was taken with severe rigors, cramps returned in the extremities, the pulse grew tremulous, the head was drawn backward, with great pain and restlessness, which continued until about two hours before she died, on the morning of the fourth day. When the restlessness ceased, we and all of the family knew she was dying. She lay quiet, and apparently free from pain, talked of death, and seemed to realize that this change was death coming upon her. The pulse gradually failed at the wrist, coma set in, and we expected in a few minutes death would close the scene. What was our surprise, the womb began to contract, the mouth quickly opened, and without apparent will or consciousness of the mother, a child was born alive. The placenta came naturally away, and the womb resumed its normal size. After some minutes, the mother opened wide her eyes, and, seeing by the anxious faces of those around that something had happened, asked: "What is the matter?" I told her she had just had a baby. "Is it alive?" she asked. "Yes," was the reply. She closed her eyes, and without a moan or struggle expired.

Here was life in and out of death.

This infant was taken and adopted by the grandmother, a woman over sixty years of age, who, to appease its crying, accustomed it to nurse at her dried-up breast. The milk returned. The child thrived, and is now a promising five-year-old boy.

In an experience of thirty years' practice, we have never seen so wonderful a case. *In articulo mortis* the womb delivers the child, as it were, in haste, and that child raised upon the breast of a woman over sixty years of age!

